# The impact of COVID-19 on aflibercept treatment of neovascular AMD in Sweden – data from the Swedish Macula Register

**DOI:** 10.1186/s12886-024-03326-8

**Published:** 2024-01-30

**Authors:** Isac Wickman, Monica Lövestam-Adrian, Elisabet Granstam, Ulrika Kjellström, Marion Schroeder

**Affiliations:** 1 Department of Ophthalmology, Department of Clinical Sciences Lund, Lund University, Lund, Sweden; 2 Department of Ophthalmology, Skåne University Hospital, Lund, Sweden; 3 Department of Surgical Sciences, Uppsala University, Uppsala, Sweden; 4 Department of Ophthalmology, Uppsala University Hospital, Uppsala, 751 85 Sweden

**Keywords:** Swedish Macula Register, SARS-CoV-2, Age-related macular degeneration, Neovascular AMD, COVID-19

## Abstract

**Background:**

The purpose of the study was to compare the real-world aflibercept treatment and visual outcomes, and to examine the adherence to pandemic guidelines in two groups of patients with treatment-naïve neovascular age-related macular degeneration (nAMD) before and during the first year of the COVID-19 pandemic in Sweden up to the 1-year follow-up.

**Methods:**

This is a retrospective observational study including 2915 treatment naïve eyes with nAMD. Using data from the Swedish Macula Register (SMR), 1597 eyes initiating treatment between 1 July 2018 and 31 January 2019 (pre-pandemic group) were compared with 1318 eyes starting treatment between 1 February and 31 August 2020 (pandemic group). The eyes were then followed for 1 year ± 2 months, hence the first group was unaffected by the pandemic while the second group was affected. The focus was on baseline characteristics, visual acuity (VA) change from baseline, number of injections, treatment regimen, number of appointments and the frequency and length of appointment delays. The Wilcoxon Signed-Rank Test was used to compare baseline VA to follow-up VA within the respective groups. The Mann-Whitney U-test and Fisher’s exact test were used to compare outcomes between the groups.

**Results:**

Baseline characteristics were similar between the two groups. The percentage of eyes with an available follow-up VA after 1 year was 58% in the pre-pandemic group vs. 44% in the pandemic group. VA in the pre-pandemic group had increased significantly after 1 year, from 62.2 ± 14.1 letters to 64.8 ± 16.1 letters (*n* = 921); *p* < 0.0001. In the pandemic group, VA increased from 61.1 ± 15.8 to 64.9 ± 16.9 (*n* = 575); *p* < 0.0001. There was no significant difference in mean VA change between the groups; *p* = 0.1734. The pre-pandemic group had significantly more delays than the pandemic group, 45% vs. 36%; *p* < 0.0001.

**Conclusions:**

The pre-pandemic and pandemic groups had similar VA gains at 1-year follow-up, but with a reduced number of available VA in the pandemic group. Clinics were able to implement and prioritize injection visits excluding VA measurements, helping to reduce delays and maintain VA gains during the COVID-19 pandemic.

**Supplementary Information:**

The online version contains supplementary material available at 10.1186/s12886-024-03326-8.

## Background

Age-related macular degeneration (AMD) is the main cause of blindness in the elderly population in developed countries [1, 2]. The global prevalence of AMD is 8.7%, and a steady increase is expected in the future [3]. Intravitreal administration of anti-vascular endothelial growth factor (anti-VEGF) has been shown to be an effective treatment to slow the progression of neovascular age-related macular degeneration (nAMD) and sustain visual acuity (VA) [4, 5]. Studies have indicated that the use of anti-VEGF treatment significantly reduces the incidence of legal blindness due to nAMD [6, 7]. Today, intravitreal anti-VEGF injections are the standard of care for nAMD.

The Swedish Macula Register (SMR) is a quality registry documenting visits of nAMD patients treated with intravitreal injections in ophthalmology clinics throughout Sweden. The registry represents, with about 85% coverage, a reliable source of administered intravitreal anti-VEGF injections in Sweden [8].

Since its outbreak in March of 2020, the COVID-19 pandemic had a considerable impact on global health, resulting in over 6.9 million deaths as of August 2023 [9]. While no lockdown was imposed in Sweden, there were negative implications for outpatient ophthalmology clinics throughout the country. The annual report of the SMR showed a reduced number of individual visits where treatment for nAMD was administered in Sweden during the first months of the pandemic, dropping from 9 834 to 7 301 between January and April of 2020 [8]. In April 2020, both international and Swedish recommendations were released to guide ophthalmology clinics treating macula patients during the COVID-19 pandemic. Guidelines included prioritizing patients at higher risk of permanent vision loss, as well as spacing out appointments to reduce the risk of spread in waiting rooms and prioritizing injection visits ahead of VA visits in order to reduce patients’ time spent in the clinic and hence reduce the risk of spreading and contracting COVID-19 [10, 11]. COVID-19 lockdowns internationally resulted in vision loss for eyes with nAMD [12–15]. A study by Barequet et al. 2023, however, did not show worse VA results in nAMD patients treated during COVID-19 lockdowns compared with patients treated pre-COVID-19 despite a reduced number of intravitreal injections given in the former group [16]. To the knowledge of the authors, a study outlining the effects of COVID-19-induced delays of anti-VEGF injections on a Swedish population of AMD patients has not been published before.

The purpose of the present study was to analyse data from the SMR to explore the extent to which the COVID-19 pandemic affected the results of anti-VEGF treatment and visual outcomes of nAMD patients in Sweden by comparing data for patients starting treatment before versus during the pandemic. Furthermore, we wanted to see whether the guidelines were implemented and whether that would influence visual acuity gains negatively compared to patients treated in non-pandemic circumstances.

## Methods

### Data collection and study population

This was a multi-center retrospective observational study. All patient data were extracted from the SMR, which requires patient consent ahead of registration. As of 2021, data from 45 clinics and 49 510 patients had been recorded in the registry. The Ethics Board of Lund University approved the study protocol, and the Tenets of the Declaration of Helsinki were followed when conducting the present study.

Eyes were divided into two groups. The “pre-pandemic” group consisted of 1597 eyes having their first visit between 1 July 2018 and 31 January 2019, while the “pandemic” group included 1318 eyes with a first visit between 1 February and 31 August 2020. This means that the pre-pandemic group was unaffected by the COVID-19 pandemic throughout the follow-up period, while the pandemic group was affected.

Inclusion criteria were a follow-up period of at least 1 year ± 2 months and treatment with aflibercept intravitreal injections at some point during the study period. Furthermore, all included eyes had to have a baseline VA value recorded. 69 eyes were excluded because they did not have a baseline VA, 795 eyes had their treatment discontinued before the 1-year follow-up and 1401 eyes had not been treated with aflibercept. Hence, 2265 (44%) of the eyes that started treatment during the inclusion period were excluded since they did not fulfill the inclusion criteria. Data extracted from the registry included age, gender, VA, symptom duration, type of membrane, diagnostic method, type of drug and switches between drugs, treatment regimen, number of injections, total number of appointments, number of delayed visits and number of VA examinations. The length of delay was defined as duration from when the visit was originally scheduled to when the visit occurred. VA was measured using Early Treatment Diabetic Retinopathy Study (ETDRS) letters. In cases where such values were not available, Snellen values were converted to ETDRS letters as per Gregori et al. 2010 [17]. Types of treatment regimen included: fixed regimen, meaning a fixed treatment interval is chosen and maintained; Treat & Extend (T&E), a proactive regimen whereby the patient receives an injection at every visit, and the injection interval is either shortened or extended depending on the status of the macula; pro re nata (PRN), a conservative approach where treatment is only given in case of active disease.

In terms of missing values, information about initial treatment regimen was missing for 148 eyes and information on regimen change from baseline was missing for 130 eyes. Furthermore, the variable “membrane type” was unavailable for 142 eyes. Diagnostic imaging method at baseline was missing for 1214 eyes, and age was missing for 1 patient. Moreover, information about minimum and maximum days between injections was missing for 21 eyes.

The primary endpoint of the study was VA change over time compared between the groups. Secondary endpoints were the comparison of baseline data, the number of injections at 1 year follow-up, drug choice including change between drugs, treatment regimen in the first year, the length of treatment intervals, the number of VA examinations during the first year, as well as the frequency of delayed injections and appointments.

Moreover, to examine how different degrees of vision loss and gain were distributed in both the pre-pandemic and the pandemic groups, seven subgroups were created based on visual acuity change from baseline and categorized into five-letter ETDRS gain or loss increments. Special consideration was given for extreme changes of more than 15 letters gained or lost.

### Statistical analysis

Baseline data were analyzed to determine frequencies and percentages of categorical variables, while mean and standard deviations were used for continuous variables. Furthermore, for VA change from baseline within groups, Wilcoxon Signed-Rank Test was used. Comparisons between groups for continuous variables were made using the Mann-Whitney U test, and comparisons between groups for categorical variables were calculated using Fisher’s exact test. A *p*-value of < 0.05 was regarded as statistically significant. All statistical analysis was performed using SAS Enterprise guide 8.2.

## Results

### Baseline characteristics

Baseline characteristics are given in Table 1. A total of 2915 treatment naïve eyes of 2725 nAMD patients were included in the study. There were no significant differences between groups with regards to baseline VA, age and gender distribution. Moreover, a significantly larger proportion of patients in the pandemic group presented with a symptom duration of less than 2 months at baseline compared with the pre-pandemic group.

**Table 1:** Comparison of baseline characteristics, drug choice and treatment regimen for pre-pandemic vs. pandemic eyes

Baseline characteristic	Both groups	Pre-pandemic	Pandemic	p
No. of eyes, n (%)	2915 (100)	1597 (55)	1318 (45)	
Gender, n (% of patients)				
Female	1708 (63)	910 (61)	798 (65)	0.0673
Mean age (SD)	78.4 (7.9)	78.6 (8)	78.2 (7.8)	0.1195
Missing, n (%)	1 (0)	1 (0)	0 (0)	
Mean ETDRS letters (SD)	62.0 (14.2)	62.5 (13.7)	61.4 (14.8)	0.0778
Symptom duration, n (% of eyes)				
0 - <2 months	1610 (55)	845 (53)	765 (58)	0.0056
2 - <4 months	587 (20)	343 (21)	244 (19)	0.0513
4–6 months	307 (11)	171 (11)	136 (10)	0.7620
> 6 months	411 (14)	238 (15)	173 (13)	0.1814
Membrane type, n (% of eyes)				
Type 1	876 (30)	439 (27)	437 (33)	0.0003
Type 2	462 (16)	277 (17)	185 (14)	0.0275
Type 3	298 (10)	160 (10)	138 (10)	0.5793
PCV	101 (3)	63 (4)	38 (3)	0.1541
Undetermined	1036 (36)	593 (37)	443 (34)	0.1057
Missing	142 (5)	65 (4)	77 (6)	
Initial drug, n (% of eyes)				
Aflibercept	2557 (88)	1428 (89)	1129 (86)	0.0022
Ranibizumab	82 (3)	43 (3)	39 (3)	0.7359
Bevacizumab	276 (9)	126 (8)	150 (11)	0.0015
Initial treatment regimen, n (% of eyes)				
T&E	2269 (78)	1274 (80)	995 (75)	0.0053
PRN	365 (13)	179 (11)	186 (14)	0.0177
Fixed	61 (2)	37 (2)	24 (2)	0.4353
Other	72 (2)	29 (2)	43 (3)	
Missing	148 (5)	78 (5)	70 (5)	

Abbreviations: ETDRS, Early Treatment Diabetic Retinopathy Study; PCV, polypoidal choroidal vasculopathy; T&E, Treat & Extend; PRN, Pro Re Nata

Imaging techniques used to diagnose nAMD for the patients included in the study were Optical Coherence Tomography (OCT), Optical Coherence Tomography Angiography (OCT-A), Flourescein Angiography (FA) and Indocyanine Green Angiography (ICG). These imaging modalities were used in various combinations. The two most frequently used imaging types at the baseline clinic visit for the whole study population was OCT (18%) and a combination of OCT and OCT-A (30%). A total of 42% of patients did not have a registered imaging type recorded at baseline.

In the pre-pandemic group that started treatment between 1 July 2018 and 31 January 2019, 921 out of 1597 eyes (58%) had an available VA value at 1-year follow-up vs. 575 out of 1318 eyes (44%) starting treatment between 1 February and 31 August 2020 in the pandemic group. There was a significant difference between the groups in the number of eyes that had a VA-value at follow up; *p* < 0.0001. Furthermore, we compared the baseline characteristics of the patients who were followed up with VA after 1 year with those of patients who did not have a follow-up value for VA. This comparison was made separately for the pre-pandemic and the pandemic groups, see Tables 2 and 3. Baseline characteristics were similar in the pre-pandemic patients with and without a follow-up VA, however percentages of patients initially given aflibercept or bevacizumab differed significantly. In the pandemic group, there were no significant difference between the follow-up and non-follow-up patients regarding gender, age, or baseline VA, however bevacizumab occurred significantly more often as the initial drug in the non-follow-up group than in the follow-up group. Supplementary Table 1 presents a comparison of baseline characteristics, drug choice and treatment regimen for the patients with an available VA at 1-year follow-up in the pre-pandemic vs. pandemic groups. Moreover, data concerning eyes either with or without a baseline VA that had their treatment discontinued before the 1-year follow-up are presented in Supplementary Table 2.

**Table 2:** Comparison of baseline characteristics and drug choice for eyes starting treatment *before* the COVID-19 pandemic

Baseline characteristic	Both groups	With 1-year VA follow-up	Without 1-year VA follow-up	p
No. of eyes, n (%)	1597 (100)	921 (58)	676 (42)	
Gender, n (% of patients)				
Female	910 (61)	513 (59)	397 (64)	0.1066
Mean age (SD)	78.6 (8)	78.9 (8)	78.2 (8)	0.0632
Missing, **n (%)**	1 (0)	1 (0)	0 (0)	
Mean ETDRS letters (SD)	62.5 (13.7)	62.2 (14.1)	62.9 (13)	0.5853
Symptom duration, n (% of eyes)				
0 - <2 months	845 (53)	501 (54)	344 (51)	0.1710
2 - <4 months	343 (21)	185 (20)	158 (23)	0.1232
4–6 months	171 (11)	96 (10)	75 (11)	0.6827
> 6 months	238 (15)	139 (15)	99 (15)	0.8313
Membrane type, n (% of eyes)				
Type 1	439 (27)	237 (26)	202 (30)	0.0672
Type 2	277 (17)	156 (17)	121 (18)	0.6382
Type 3	160 (10)	91 (10)	69 (10)	0.8658
PCV	63 (4)	41 (4)	22 (3)	0.2434
Undetermined	593 (37)	359 (39)	234 (35)	0.0798
Missing	65 (4)	37 (4)	28 (4)	
Initial drug, n (% of eyes)				
Aflibercept	1428 (89)	841 (91)	587 (87)	0.0050
Ranibizumab	43 (3)	30 (3)	13 (2)	0.1184
Bevacizumab	126 (8)	50 (5)	76 (11)	< 0.0001

Abbreviations: ETDRS, Early Treatment Diabetic Retinopathy Study; PCV, polypoidal choroidal vasculopathy

**Table 3:** Comparison of baseline characteristics and drug choice of eyes starting treatment *during* the COVID-19 pandemic

Baseline characteristic	Both groups	With 1-year VA follow-up	Without 1-year VA follow-up	p
No. of eyes, n (%)	1318 (100)	575 (44)	743 (56)	
Gender, n (% of patients)				
Female	798 (65)	348 (64)	450 (65)	0.9522
Mean age (SD)	78.2 (7.8)	78 (8.3)	78.4 (7.4)	0.5181
Mean ETDRS letters (SD)	61.4 (14.8)	61.1 (15.8)	61.6 (14)	0.9750
Symptom duration, n (% of eyes)				
0 - <2 months	765 (58)	340 (59)	425 (57)	0.4997
2 - <4 months	244 (19)	104 (18)	140 (19)	0.7749
4–6 months	136 (10)	48 (8)	88 (12)	0.0443
> 6 months	173 (13)	83 (14)	90 (12)	0.2185
Membrane type, n (% of eyes)				
Type 1	437 (33)	172 (30)	265 (36)	0.0268
Type 2	185 (14)	80 (14)	105 (14)	0.9361
Type 3	138 (10)	60 (10)	78 (10)	1.0000
PCV	38 (3)	20 (3)	18 (2)	0.3188
Undetermined	443 (34)	209 (36)	234 (31)	0.0640
Missing	77 (6)	34 (6)	43 (6)	
Initial drug, n (% of eyes)				
Aflibercept	1129 (86)	502 (87)	627 (84)	0.1537
Ranibizumab	39 (3)	20 (3)	19 (3)	0.3312
Bevacizumab	150 (11)	53 (9)	97 (13)	0.0356

Abbreviations: ETDRS, Early Treatment Diabetic Retinopathy Study; PCV, polypoidal choroidal vasculopathy

### Mean change in visual acuity

Out of those with an available follow-up ETDRS value in the pre-pandemic group, VA increased significantly from 62.2 ± 14.1 letters at baseline to 64.8 ± 16.1 letters, hence the change was 2.6 ± 13.6 letters; *p* < 0.0001. In the pandemic group, VA also increased significantly from 61.1 ± 15.8 at baseline to 64.9 ± 16.9, showing an increase of 3.8 ± 16.3 letters; *p* < 0.0001. No significant difference was found when comparing the changes in VA between the two groups; *p* = 0.1734.

**Fig. 1:**
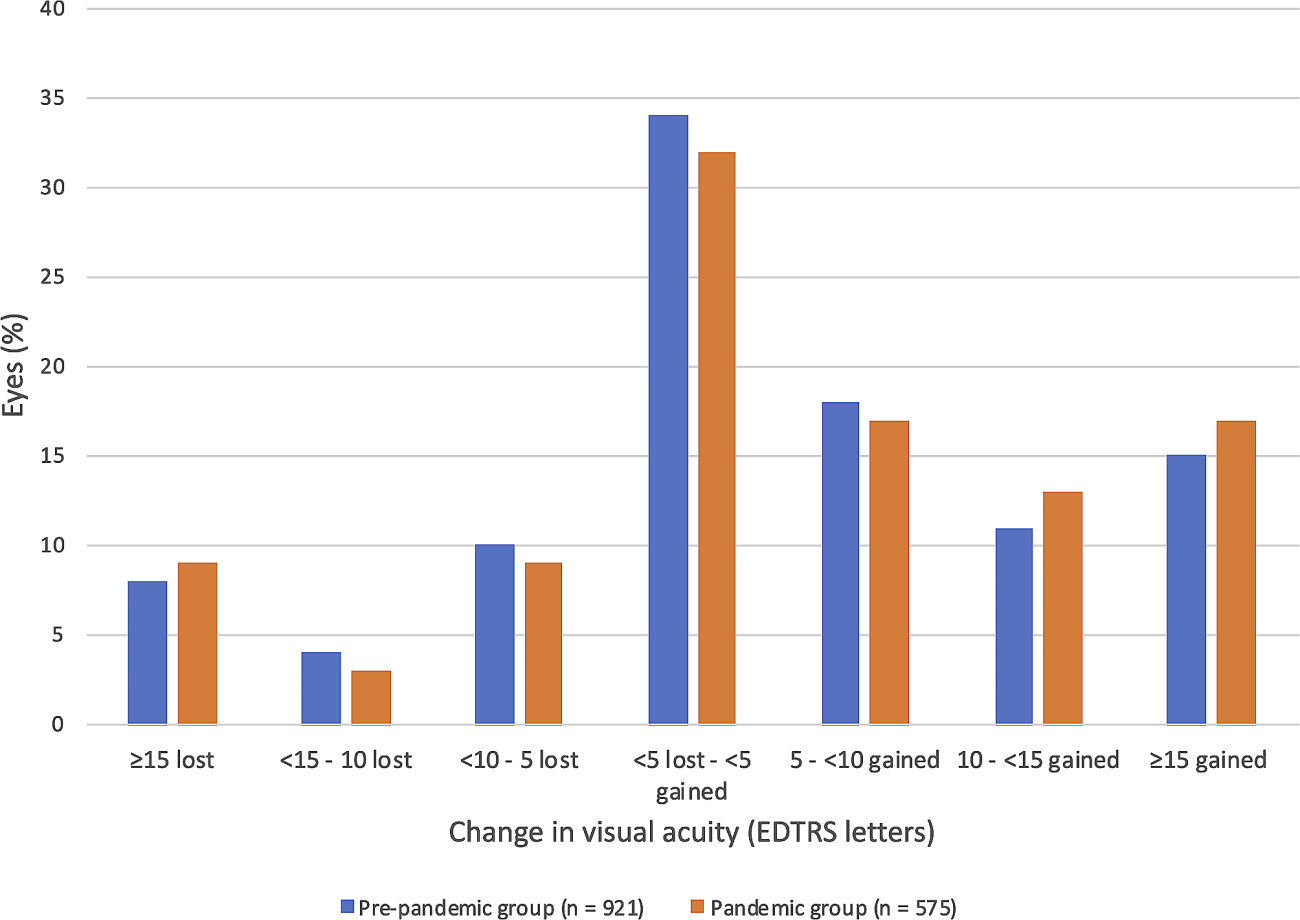
One year change in visual acuity from baseline in pre-pandemic vs. pandemic eyes. (Percentage of eyes that gained and lost VA up to year 1 for patients followed for at least 1 year ± 2 months, stratified into 7 groups depending on many letters they gained or lost)

Furthermore, eyes in both the pre-pandemic and pandemic groups were divided into 7 subgroups depending on how many letters they lost or gained, as shown in Fig. 1. There was no statistically significant difference in how the percentages of eyes were distributed between the different categories of ETDRS letters gained or lost for both the pre-pandemic and the pandemic groups followed-up to year one (Fig. 1).

### Number of injections and type of drug

The mean number of injections given to each eye during the follow-up year was statistically significantly different between the groups, with a mean of 7.4 ± 2.2 total injections in the pre-pandemic group vs. 7.5 ± 2.3 in the pandemic group; *p* = 0.0063. The distribution of the different types of anti-VEGF drugs was the following: in the pre-pandemic group, a mean of 6.7 ± 2.6 aflibercept injections were administered per eye vs. 6.7 ± 2.7 in the pandemic group; *p* = 0.2963. In the pre-pandemic group, a mean 0.5 ± 1.7 bevacizumab injections were given per eye vs. 0.7 ± 1.8 in the pandemic group, differing significantly; *p* = 0.0003. Lastly, a mean of 0.2 ± 1.0 ranibizumab injections were given to eyes in both the pre-pandemic and the pandemic groups; *p* = 0.0705.

### Treatment regimen

As seen in Table 1, the main initial treatment regimen for eyes in both groups was T&E. In terms of changing from their baseline regimen to another, 36% (*n* = 570) of all eyes in the pre-pandemic group changed treatment regimen within one year from baseline. On the other hand, that figure was significantly lower at 19% (*n* = 255) for eyes in the pandemic group; *p* < 0.0001.

For eyes in both the pre-pandemic and pandemic groups starting on a T&E regimen, the distribution of eyes switching to different regimens was as follows: 25% (*n* = 319) of eyes that started on a T&E schedule before COVID-19 changed their regimen during the first year, while that number was significantly lower at 7% (*n* = 70) for T&E eyes during the pandemic; *p* < 0.0001. Out of the eyes that started with T&E and then switched to another regimen, 1% (*n* = 2) of eyes in the pre-pandemic group switched to a fixed regimen vs. 6% (*n* = 4) in the pandemic group; *p* = 0.0109. Furthermore, most eyes in both groups switched to PRN after starting with T&E: 67% (*n* = 214) in the pre-pandemic group vs. 63% (*n* = 44) in the pandemic group; *p* = 0.4891.

Regarding the eyes starting on a PRN regimen at baseline and switching to another regimen, this was the distribution: 73% (*n* = 130) vs. 43% (*n* = 80) switched regimen in the pre-pandemic and pandemic groups, respectively; *p* < 0.0001. Out of these eyes, 89% (*n* = 116) in the pre-pandemic group vs. 88% (*n* = 70) in the pandemic group changed regimen from PRN to T&E, *p* = 0.8237. A small proportion, 2% (*n* = 3) vs. 1% (*n* = 1) switched to a fixed regimen in the pre-pandemic and pandemic groups, respectively; no significant difference was found.

Out of all eyes that changed their treatment regimen, the percentage that switched to a fixed regimen at some point during the first year was similar in both groups; 4% (*n* = 21) in the pre-pandemic group vs. 5% (*n* = 12) in the pandemic group; *p* = 0.5642.

### Number of clinic visits and VA examinations

It was significantly observed that consultations during the pandemic were more frequently associated with intravitreal injection therapy (IVT) appointments that excluded VA testing, and that a smaller proportion of VA tests overall were reported during these consultations. The total number of registered clinic visits was 12 783 in the pre-pandemic group, and 10 585 in the pandemic group. The percentage of those visits that included VA testing was 52% (*n* = 6661) and 44% (*n* = 4661), respectively; *p* < 0.0001. Moreover, in 92% (*n* = 11 779) of visits in the pre-pandemic group, an anti-VEGF injection was administered. That figure was 94% (*n* = 9 958) in the pandemic group, differing significantly; *p* < 0.0001. When comparing visits where injections were given and VA was not tested, those made up 48% (*n* = 6103) in the pre-pandemic group and 56% (*n* = 5898) in the pandemic group; *p* < 0.0001.

In the first year, the mean number of VA examinations were 4.2 ± 1.5 in the pre-pandemic group and 3.5 ± 1.6 in the pandemic group; *p* < 0.0001.

### Treatment interval and delayed appointments

In the pre-pandemic group, the mean duration between baseline visits and initiation of anti-VEGF treatment was 4.1 ± 18.5 days vs. 5.2 ± 23.2 days; *p* < 0.0001 in the pandemic group. In terms of treatment interval, there was a similar number of mean minimum days between injections in both groups, with the pre-pandemic group having a mean 28.7 ± 13 days vs. 29.9 ± 21.7 in the pandemic group; *p* = 0.1882. The mean maximum days between injections were 87.8 ± 40.6 days vs. 85.7 ± 43.5 days; *p* = 0.0025.

Out of the total recorded number of clinic visits, information about whether there was a delay was provided for 88% (*n* = 11 186) of the clinic visits in the pre-pandemic group vs. 88% (*n* = 9 267) of visits in the pandemic group. Delayed appointments included follow-up appointments as well as injections. In the pre-pandemic group, 45% (*n* = 5056) of visits were delayed during the first year. In the pandemic group, there were significantly fewer delays, with 36% (*n* = 3301) of visits being postponed; *p* < 0.0001. The length of delay was similar in both groups, with the mean delayed time being 13.8 ± 22.6 days for the pre-pandemic group and 13.8 ± 25.8 days for the pandemic group; *p* = 0.0643.

## Discussion

The present study compared two groups of nAMD patients starting anti-VEGF treatment before and during the COVID-19 pandemic, up to the 1-year point. In terms of baseline characteristics, the two groups showed a similar distribution of age, gender, and VA, confirming that the same type of nAMD patients got access to medical care during the pandemic compared to before it started. There were, however, significant differences regarding symptom duration, with a larger proportion of the pandemic group having a symptom duration shorter than 2 months, indicating that despite appointments being restricted due to the pandemic, pandemic patients did not have to wait longer than pre-pandemic patients from onset of symptoms to their first clinic visit. It could be debated that the 2-month range is rather large and does not show a nuanced picture of symptom duration, and hence its clinical relevance could be questioned. Furthermore, the percentage of type 1 macular neovascularization (MNV) prevalence was significantly higher in the pandemic group than in the pre-pandemic group, while the relationship was inversed for type 2 MNV. A study by Jiang et al. 2021 linked type 1 MNV to a higher chance of remaining stable during treatment interruption in the COVID-19 period, hence lesion type could be a potential contributing factor to VA maintenance [18].

Regarding the diagnostic imaging methods performed to diagnose nAMD, the results indicate a more widespread use of non-invasive imaging methods as well as a trend towards using OCT-A in addition to OCT to aid in diagnosis of nAMD, which could be due to its non-invasive nature and ability to accurately assess MNV lesions [19, 20]. Another reason for choosing OCT and OCT-A could be that they are less time-consuming methods than FA and ICG. An FA procedure can take up to 20 min, compared to OCT and OCT-A which are normally finished within a few minutes. This means that the type of imaging chosen is important since longer time spent with the patient means more potential exposure to COVID-19, hence the choice of diagnostic imaging at baseline could potentially have helped clinics adhere to the COVID-19 recommendations. We should, however, consider the fact that there were many patients where the diagnostic method used to diagnose nAMD had not been registered. The high number of missing data could possibly be explained by the fact that the variable “Diagnostic method” is optional in the SMR. However, there should not be any differential information bias between the two periods.

The present study showed that 58% of eyes in the pre-pandemic group vs. 44% of eyes in the pandemic group had a registered follow-up VA after 1 year. Furthermore, there were significantly more clinic visits that included VA testing in the pre-pandemic group. This indicates that VA examinations were not prioritized to the same extent for patients starting treatment during the pandemic. This is further supported by the fact that 56% of first-year appointments in the pandemic group were injection visits that excluded VA testing, while that number was significantly lower at 48% for the pre-pandemic group. Minimizing VA examinations in favor of injection appointments was in line with both international and national guidelines [10, 11]. It should be noted, however, that the SMR does not specify whether the injection appointment included an OCT image or not. In cases where an OCT was taken as well, time for patients in contact with clinic staff might potentially have increased with a couple of minutes. Another reason for lower attendance to less prioritized appointments such as those with VA examinations during the pandemic may be the fact that the main patient cohort affected by AMD, the elderly, is a risk group for severe COVID-19 complications [21–23]. Hence, cancellations for follow-up appointments might have been made by the patients themselves. Another explanation might be clinics reducing the time patients spend in the clinic to lower the risk of spread. In the above-mentioned guidelines for treatment of macular patients during the COVID-19-pandemic, it was also recommended that providers should switch to a fixed regimen to reduce the need for OCT and VA appointments [10]. Further reasons for reduced appointments were clinic staff being required to stay at home in cases of symptoms of upper respiratory tract infection or confirmed COVID-19 diagnosis, as well as being relocated to COVID wards due to the high patient burden there.

Interestingly, delayed visits were significantly more frequent in the pre-pandemic group. A possible explanation for this could be that during the pandemic period, patients and medical staff were more attentive to the necessity of strict follow-up and treatment protocols for ensuring optimal functioning and planned patient visit schedules within healthcare facilities. More delayed visits for the pre-pandemic group, coupled with the fact that the pandemic group registered a smaller fraction of VA visits, and that visits that included injections but excluded VA measurements made up a significantly larger portion of visits in the pandemic group than in the pre-pandemic group, indicates that the clinics were able to provide anti-VEGF treatment to the pandemic group successfully by prioritizing appointments that did not include VA measurement. Another sign of successful prioritization during the pandemic could be the adherence to a T&E regimen. While there was a significant difference between the groups, with 80% of the pre-pandemic group starting with T&E vs. 75% in the pandemic group, the main treatment regimen at baseline was T&E for both cohorts. Moreover, 25% of patients in the pre-pandemic group that started with T&E, changed to another regimen at some point during the follow-up period. However, that number was significantly lower at 7% for the pandemic group. A possible impact of continuing with a T&E regimen during the first year of treatment could be that the pandemic group was able to maintain their VA without needing repeated follow-up visits, which would have been the case if they had switched to a PRN regimen. The reduced fraction of VA appointments in the pandemic group paired with the fact that they maintained VA just as well as the pre-pandemic group, indicates that it was a successful strategy. In addition to using less time-consuming diagnostic imaging, this is a potential way of reducing time in clinic and hence COVID-19 exposure. Jiang et al. 2021 provided support for the T&E schedule during COVID-19, by showing that patients on a T&E regimen remained more stable than those on a PRN regimen in terms of VA outcome [18]. Furthermore, the T&E regimen has yielded good VA results in previous studies, showing a superiority towards the PRN regimen [24–26]. With regards to injections, the mean number of injections administered per eye in a 1-year-period was statistically significantly larger in the pandemic group compared to in the pre-pandemic group. However, while statistically significant, the small difference most likely will have no clinical significance.

Early initiation of anti-VEGF treatment has previously been shown to increase the likelihood of better VA outcomes in nAMD patients [27]. In the present study, most patients in both groups presented with a symptom duration of less than 2 months at baseline.

In terms of VA change, no significant difference was detected between the two groups. The results could be impacted by the fact that only 58% of patients in the pre-pandemic group and 44% of patients in the pandemic group had a registered follow-up VA after 1 year. The VA gain in both groups was slightly higher when comparing with another real-world study by Ciulla et al. 2020, which showed a mean gain of 1 letter after 1 year [28]. Moreover, our results were similar to those of Holz et al. 2015, whose study showed a VA gain of 2.4 letters at the 1-year point [29]. Hence, the results of the present study indicate that it is possible to maintain similar VA gains despite the difficulties of treating patients during a pandemic, such as increased cancellation of appointments as well as the need to adhere to guidelines advocating significantly reduced time for patients in clinic. When comparing with randomized controlled trials, however, the VA gain in our study was relatively low. In the VIEW study, mean VA gain after 1 year was 8.3 to 9.3 letters for patients receiving aflibercept. Furthermore, patients receiving ranibizumab treatment in the MARINA and ANCHOR studies showed a mean 6.5 to 7.2 letter gain vs. an 8.5 to 11.3 letter gain at 52 weeks [4, 5, 30]. However, the baseline VA in the present study was higher in comparison with the clinical trials. Baseline VA in the MARINA and VIEW studies ranged from 53.1 to 53.7 letters and 53.6 to 54.0 letters, respectively, while baseline VA in the present study was 62.2 letters for the pre-pandemic group and 61.1 letters for the pandemic group. This contrast of up to nearly 10 letters might indicate the occurrence of a ceiling effect in the present study, which would explain why VA gain was lower in comparison. The ceiling effect, however, is normally attributed to eyes with a baseline VA of > 70 letters, which indicates that it might not be the sole explanation for the difference in VA gain between the present study and the clinical trials [31]. In any case, there is often a tendency for VA results in real-world studies to be inferior compared to in clinical trials, where strict treatment intervals and follow-ups are applied.

The present study showed better pandemic VA results than several studies conducted on nAMD patients in countries where there were COVID-19-induced lockdowns. Zarranz-Ventura et al. 2022 showed a reduction in VA for eyes treated for nAMD spanning from − 0.4 to -3.8 logarithm of the minimum angle of resolution (logMAR) letters [12]. Furthermore, a study by Stattin et al. 2022 indicated a significant VA deterioration of 2.5 ± 6 letters for nAMD when comparing VA before COVID-19 lockdown and after [14]. Moreover, Yeter et al. 2021 showed a significant VA loss from 0.67 logMAR to 0.78 logMAR after lockdown. VA recovered to 0.69 logMAR after restarting treatment but did not change significantly from baseline [15]. Still, these studies are not fully comparable to the present study. This is because they were conducted in countries that imposed a national lockdown, hence leading to involuntary interruption of treatment. Meanwhile Sweden’s COVID-19 response was, at its peak, of a significantly less restrictive nature [32]. This is a likely explanation for the fact that Swedish eyes did not significantly worsen in terms of visual outcome, since it not only allowed for easier access to healthcare, but also might have affected the attitudes of patients towards visiting the clinic positively. It is important to mention, however, that Barequet et al. 2023 provided similar results to the present study, showing that there was no significant difference in VA change between eyes treated before, during or after the pandemic despite a national lockdown [16]. The authors of that study suggested that reasons for that might have been that the lockdown of different countries could have been of varying strictness and durations, but also because most of their patients were on a T&E regimen. As was mentioned earlier in this discussion section, T&E might have been a factor for the pandemic group maintaining VA in the present study. Hence, the results of Barequet et al. 2023 are important as they suggest that results can be maintained despite a lockdown, possibly through having patients on a T&E regimen.

There are limitations to the present study. Due to the retrospective study design, there were missing values. The percentage of patients with a follow-up VA at the 1-year point was 58% in the pre-pandemic group vs. 44% in the pandemic group. In the pandemic group, this low number might partly be explained by the clinics reducing the number of VA examinations. However, despite there being a significantly larger percentage of patients with a 1-year follow-up VA in the pre-pandemic group, both groups had many missing 1-year VA results. One explanation for the discrepancy between the pre-pandemic and the pandemic groups could be that patients in the pre-pandemic group who started in January 2019 could possibly have had their follow-up scheduled in March of 2020, hence meaning that COVID-19 restrictions postponed those appointments. As shown in Tables 2 and 3, the patients who had and those who did not have a follow-up VA registered at the 1-year follow-up were homogenous with regards to many baseline features in both comparisons. Hence baseline characteristics might not be able to explain why such a large number of patients were missing a 1-year follow-up. For future studies on COVID-19 and its effects on nAMD treatment, we propose interviewing the departments that register data into the SMR. This might give further insight into how prioritization of appointments was carried out and could help explain alterations in the choice of diagnostic imaging and the number of delays.

As shown in Tables 2 and 3, bevacizumab was a more common drug choice at baseline in the loss-to-follow-up groups before and during the pandemic. This raises the question of whether there are certain characteristics in this subgroup of patients explaining their reduced adherence to follow-ups. In a study by van Asten et al. 2018, bevacizumab was found to be the most cost-effective anti-VEGF drug used for AMD treatment [33]. Furthermore, a study by Gower et al. 2017 showed that patients who started nAMD treatment with bevacizumab were less likely to live in high median income zipcodes than those initially given ranibizumab, for example [34]. Hence, one could argue that the variation in anti-VEGF drugs administered in our study could be explained by such socio-economic factors. In Sweden, however, all anti-VEGF agents for treating nAMD are reimbursed and come at the same low cost for the patient. Therefore, rather than attributing the variation in initial drug choice to factors related to the individual patients, it’s possible that it could be largely explained by the fact that different regions and clinics in Sweden have regional variation in procurement. For example, in 2020, Sunderby hospital used bevacizumab almost exclusively, while Falu hospital almost always used aflibercept [35].

Regarding the initial injection types in our present study, the majority of eyes in the total study sample received aflibercept. The distribution of initial drug types was aflibercept at 88%, bevacizumab at 9% and ranibizumab at 3%. This is a ratio comparable to that of what was reported in the SMR, showing a distribution of total injections given throughout 2020 across Sweden with aflibercept at 72%, bevacizumab at 22% and ranibizumab at 6% [36]. The authors are aware that there seems to be a skew towards the use of aflibercept in the present study. This could be due to the inclusion criterion that eyes had to be treated with aflibercept at some point during the study, hence this is a potential source of selection bias. However, it was chosen as an inclusion criterion since it is the most used drug in the SMR, and thus the confounding effect of using various drugs was reduced. Moreover, the distribution of drugs used in the present study is close to what was reported nationally. Still, it should be emphasized that while the study sample might reflect practice in Sweden, countries like Finland have a drastically different distribution, where annual bevacizumab rates of 80–85% have been reported [37]. Hence generalization to such countries might not be fully accurate.

The results of the present study highlight the importance of VA follow-ups; without them it is difficult to study visual outcomes and draw conclusions in a real-world setting. Therefore, a potential modification of pandemic-adjusted guidance could be to recommend more VA follow-ups. For example, it could be beneficial for patients to include VA examinations at baseline, after the loading dose of 3 injections and then yearly or when treatment-resistance is suspected. This would be a feasible guideline as VA testing does not require much time in contact with the patient, furthermore the risk of spread would be further lowered using personal protective equipment (PPE). Real-world study results are important to evaluate the impact of new guidelines, their implementation, and the follow-up of their outcomes, especially in unique situations like the COVID-19 pandemic as they can lead the way for future handling of similar situations.

## Conclusions

In conclusion, the present study showed similar significant gains of VA in both groups at 12-month follow-up for patients initiating treatment before and during the COVID-19 pandemic. Furthermore, it was found that intravitreal treatment was prioritized ahead of VA examinations, indicating that clinics acted in line with the guidelines in order to adhere to social distancing and to minimize the time spent by patients in the clinic, in turn reducing infection with COVID-19. However, low numbers of follow-up VA results at the 1-year point for both groups indicated that there is a need to further prioritize 1-year follow-ups. Contrary to what might have been expected, we found fewer delays of visits in the pandemic group. The present study demonstrates that it was possible to maintain pre-pandemic VA gains for aflibercept treated nAMD patients during the first year of the COVID-19 pandemic in Sweden. However, further studies with longer follow-up periods are needed to evaluate the long-term effects of COVID-19 on nAMD treatment.

### Electronic supplementary material

Below is the link to the electronic supplementary material.

Supplementary Material 1

## Data Availability

The datasets used and analysed during the current study are available from the corresponding author on reasonable request.

## Abbreviations

AMD: Age-related macular degeneration
Anti-VEGF: Anti-vascular endothelial growth factor
nAMD: Neovascular age-related macular degeneration
VA: Visual acuity
SMR: Swedish Macula Register
ETDRS: Early Treatment Diabetic Retinopathy Study
T&E: Treat and extend
PRN: Pro re nata
OCT: Optical Coherence Tomography
OCT-A: Optical Coherence Tomography Angiography
FA: Fluorescein Angiography
ICG: Indocyanine Green Angiography
MNV: Macular Neovascularization
PCV: Polypoidal choroidal vasculopathy
IVT: Intravitreal injection therapy
LogMAR: Logarithm of the Minimum Angle of Resolution
PPE: Personal protective equipment
