# Supplementary material for: The impact of COVID-19 on aflibercept treatment of neovascular AMD in Sweden – data from the Swedish Macula Register
Source: BMC Ophthalmol. 2024 Jan 30;24:49. doi: 10.1186/s12886-024-03326-8 (PMC10826194; doi:10.1186/s12886-024-03326-8)
Supplement: Supplementary file 1 — Supplementary Material 1 [file 12886_2024_3326_MOESM1_ESM.docx]

**Supplementary table 1: Comparison of baseline characteristics, drug choice and treatment regimen for patients with an available VA at 1-year follow-up in the pre-pandemic vs. pandemic groups**

| **Baseline characteristic** | **Both groups** | **Pre-pandemic** | **Pandemic** | **p** |
| --- | --- | --- | --- | --- |
| No. of eyes, n (%) | 1496 | 921 | 575 |  |
| Gender, n (% of patients) |  |  |  |  |
| Female | 861 (61) | 513 (59) | 348 (64) | 0.0631 |
| Mean age (SD) | 78.5 (8.1) | 78.9 (8.0) | 78.0 (8.3) | 0.0304 |
| Missing, n (%) | 1 (0) | 1 (0) | 0 (0) |  |
| Mean ETDRS letters (SD) | 61.8 (14.8) | 62.2 (14.1) | 61.1 (15.8) | 0.3391 |
| Symptom duration, n (% of eyes) |  |  |  |  |
| 0 - <2 months | 841 (56) | 501 (54) | 340 (59) | 0.0772 |
| 2 - <4 months | 289 (19) | 185 (20) | 104 (18) | 0.3470 |
| 4 – 6 months | 144 (10) | 96 (10) | 48 (8) | 0.2073 |
| >6 months | 222 (15) | 139 (15) | 83 (14) | 0.7651 |
| Membrane type, n (% of eyes) |  |  |  |  |
| Type 1 | 409 (27) | 237 (26) | 172 (30) | 0.0465 |
| Type 2 | 236 (16) | 156 (17) | 80 (14) | 0.1637 |
| Type 3 | 151 (10) | 91 (10) | 60 (10) | 0.6579 |
| PCV | 61 (4) | 41 (4) | 20 (3) | 0.4217 |
| Undetermined | 568 (38) | 359 (39) | 209 (36) | 0.4691 |
| Missing | 71 (5) | 37 (4) | 34 (6) |  |
| Initial drug, n (% of eyes) |  |  |  |  |
| Aflibercept | 1343 (90) | 841 (91) | 502 (87) | 0.0141 |
| Ranibizumab | 50 (3) | 30 (3) | 20 (3) | 0.8827 |
| Bevacizumab | 103 (7) | 50 (5) | 53 (9) | 0.0062 |
| Initial treatment regimen, n (% of eyes) |  |  |  |  |
| T&E | 1175 (79) | 751 (82) | 424 (74) | 0.1749 |
| PRN | 176 (12) | 99 (11) | 77 (13) | 0.1055 |
| Fixed | 28 (2) | 17 (2) | 11 (2) | 0.0106 |
| Other | 35 (2) | 11 (1) | 24 (4) |  |
| Missing | 82 (5) | 43 (5) | 39 (7) |  |

Abbreviations: ETDRS, Early Treatment Diabetic Retinopathy Study; PCV, polypoidal choroidal vasculopathy; T&E, Treat & Extend; PRN, Pro Re Nata

**Supplementary table 2: Comparison of baseline characteristics, drug choice and treatment regimen for non-completers in the pre-pandemic vs. pandemic groups**

| **Baseline characteristic** | **Both groups** | **Pre-pandemic** | **Pandemic** | **p** |
| --- | --- | --- | --- | --- |
| No. of eyes, n (%) | 795 (100) | 394 (50) | 401 (50) |  |
| Gender, n (% of patients) |  |  |  |  |
| Female | 476 (62) | 241 (62) | 235 (61) | 0.6574 |
| Mean age (SD) | 80.0 (9.5) | 80.4 (9.5) | 79.7 (9.6) | 0.2145 |
| Mean ETDRS letters (SD) | 52.6 (18.7) | 50.9 (18.5) | 54.3 (18.9) | 0.0071 |
| Missing, n (%) | 23 (3) | 5 (1) | 18 (4) |  |
| Symptom duration, n (% of eyes) |  |  |  |  |
| 0 - <2 months | 397 (50) | 188 (48) | 209 (52) | 0.2281 |
| 2 - <4 months | 166 (21) | 88 (22) | 78 (19) | 0.3377 |
| 4 – 6 months | 104 (13) | 47 (12) | 57 (14) | 0.3461 |
| >6 months | 128 (16) | 71 (18) | 57 (14) | 0.1490 |
| Membrane type, n (% of eyes) |  |  |  |  |
| Type 1 | 181 (23) | 74 (19) | 107 (27) | 0.0081 |
| Type 2 | 141 (18) | 73 (19) | 68 (17) | 0.5746 |
| Type 3 | 64 (8) | 31 (8) | 33 (8) | 0.8965 |
| PCV | 24 (3) | 13 (3) | 11 (3) | 0.6823 |
| Undetermined | 326 (41) | 173 (44) | 153 (38) | 0.0880 |
| Missing | 59 (7) | 30 (8) | 29 (7) |  |
| Initial drug, n (% of eyes) |  |  |  |  |
| Aflibercept | 731 (92) | 364 (92) | 367 (92) | 0.6968 |
| Ranibizumab | 16 (2) | 10 (3) | 6 (1) | 0.3236 |
| Bevacizumab | 48 (6) | 20 (5) | 28 (7) | 0.2982 |
| Initial treatment regimen, n (% of eyes) |  |  |  |  |
| T&E | 532 (67) | 269 (68) | 263 (66) | 0.1749 |
| PRN | 156 (20) | 67 (17) | 89 (22) | 0.1055 |
| Fixed | 37 (5) | 26 (7) | 11 (3) | 0.0106 |
| Other | 29 (4) | 7 (2) | 22 (5) |  |
| Missing | 41 (5) | 25 (6) | 16 (4) |  |

Abbreviations: ETDRS, Early Treatment Diabetic Retinopathy Study; PCV, polypoidal choroidal vasculopathy; T&E, Treat & Extend; PRN, Pro Re Nata
